# Supplementary material for: A genome assembly for Orinus kokonorica provides insights into the origin, adaptive evolution and further diversification of two closely related grass genera
Source: Commun Biol. 2023 Dec 2;6:1223. doi: 10.1038/s42003-023-05620-5 (PMC10693610; doi:10.1038/s42003-023-05620-5)
Supplement: Supplementary file 2 — Supplementary Information [file 42003_2023_5620_MOESM2_ESM.docx]

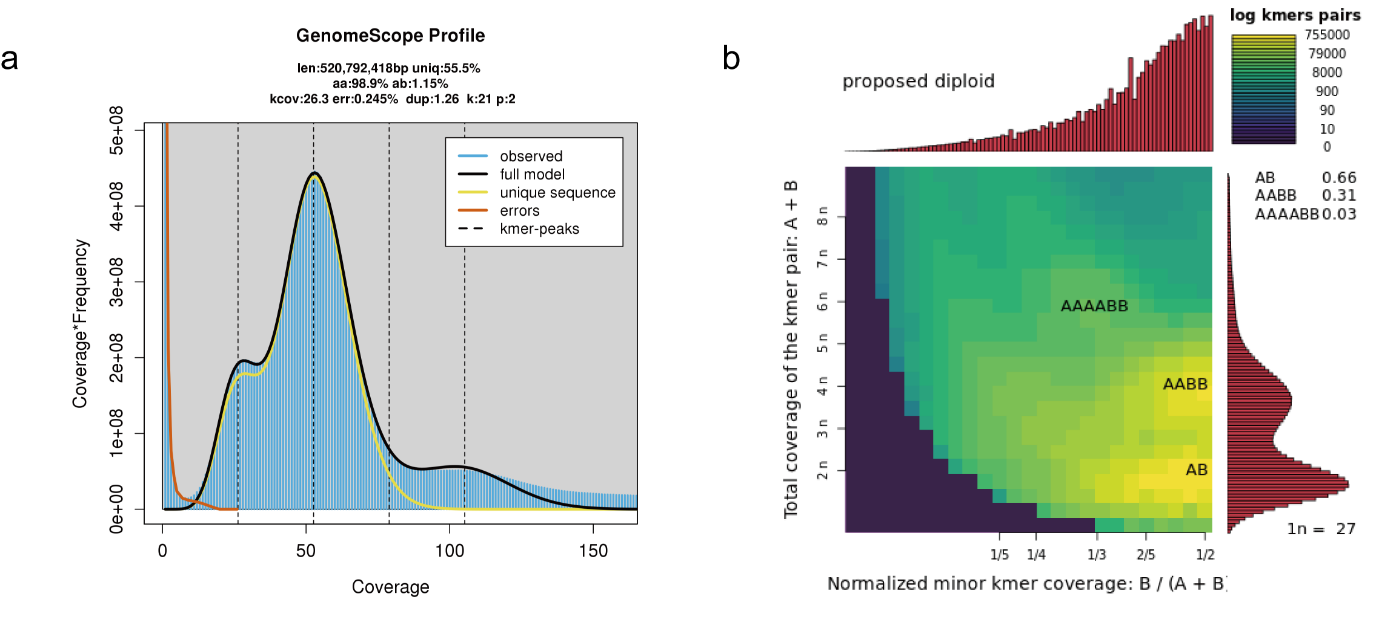


**Supplementary Figure 1** Genome characterization of *O. kokonorica*. **a** GenomeScope Profile result by using *K*-mer = 21. **b** Smudgeplot result by using *K*-mer = 27.


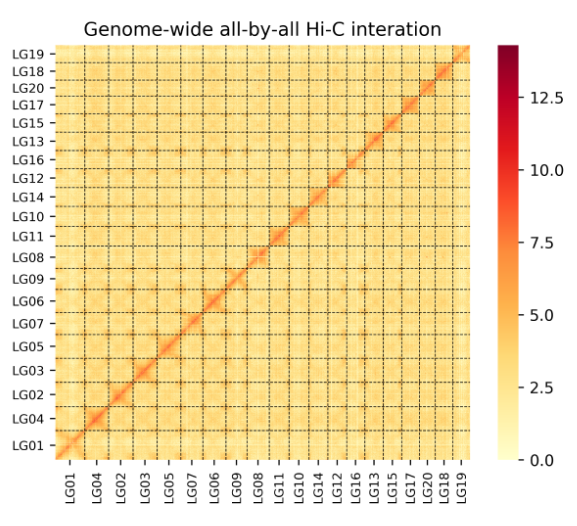


**Supplementary Figure 2** Hi-C assisted assembly of *O. kokonorica* pseudochromosomes. Heatmap showing Hi-C interactions under a resolution of 100 kb, and the antidiagonal pattern for the intrachromosomal interactions may reflect the Rabl configuration of chromatins.


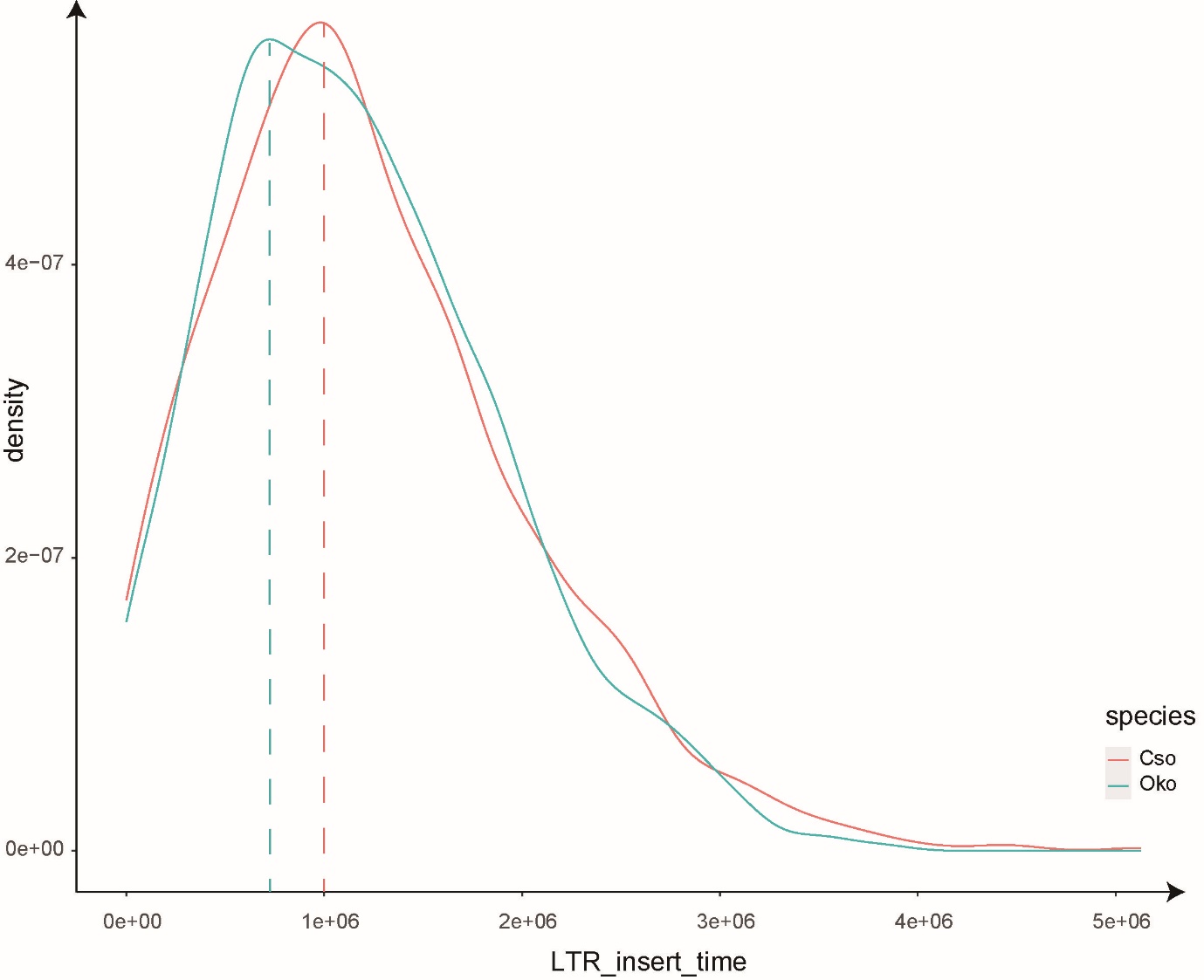


**Supplementary Figure 3** Estimated insertion time of long terminal repeat (LTR) retrotransposons in Oko (*O. kokonorica*) and Cso (*C. songorica*). The x-axis represents insertion time of the LTR-RT.


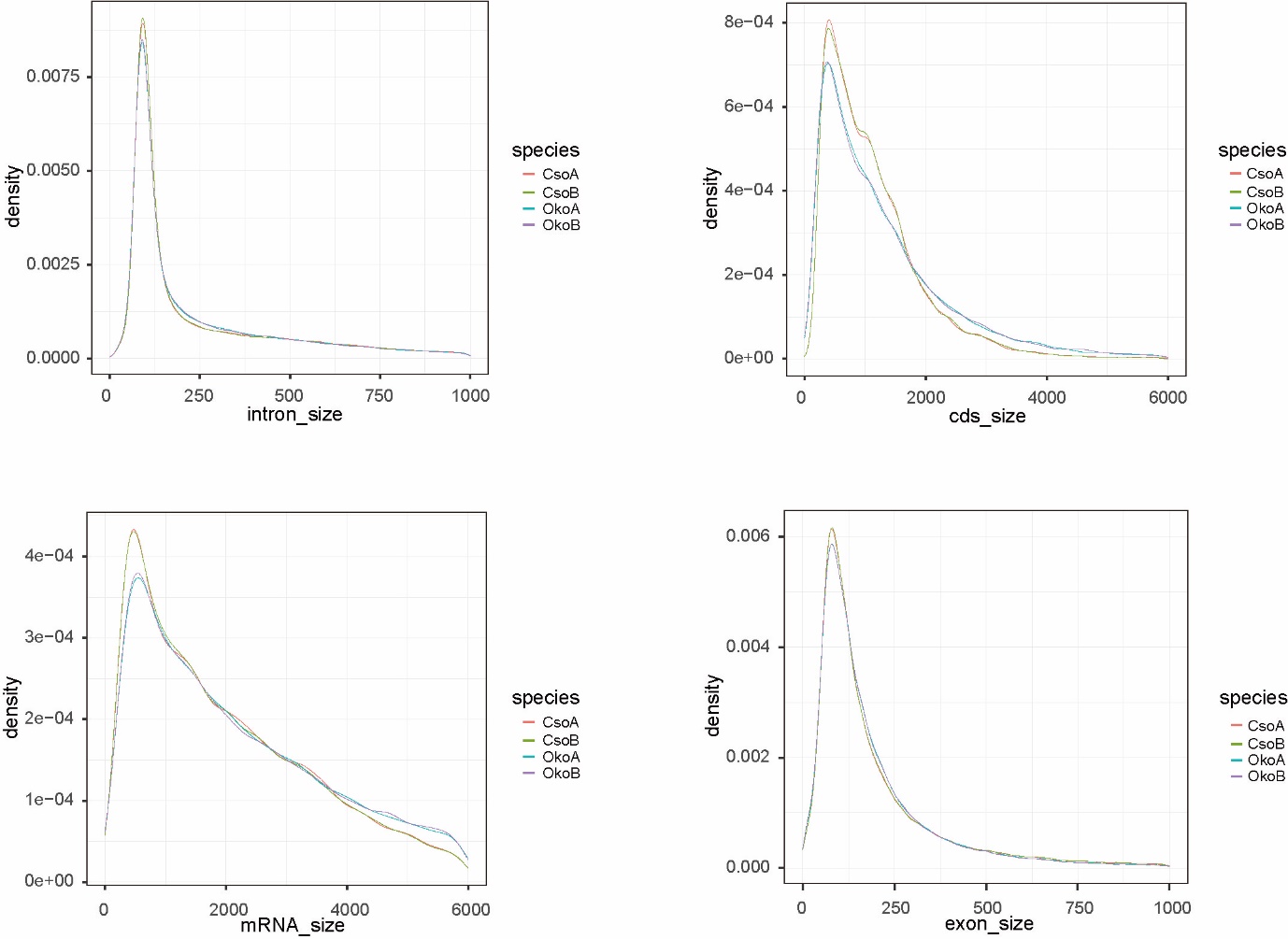


Supplementary Figure 4 Comparisons of gene structural parameters (the number and length of exons, intron length) among the subgenomes of Oko (*O. kokonorica*) and Cso (*C. songorica*).

**
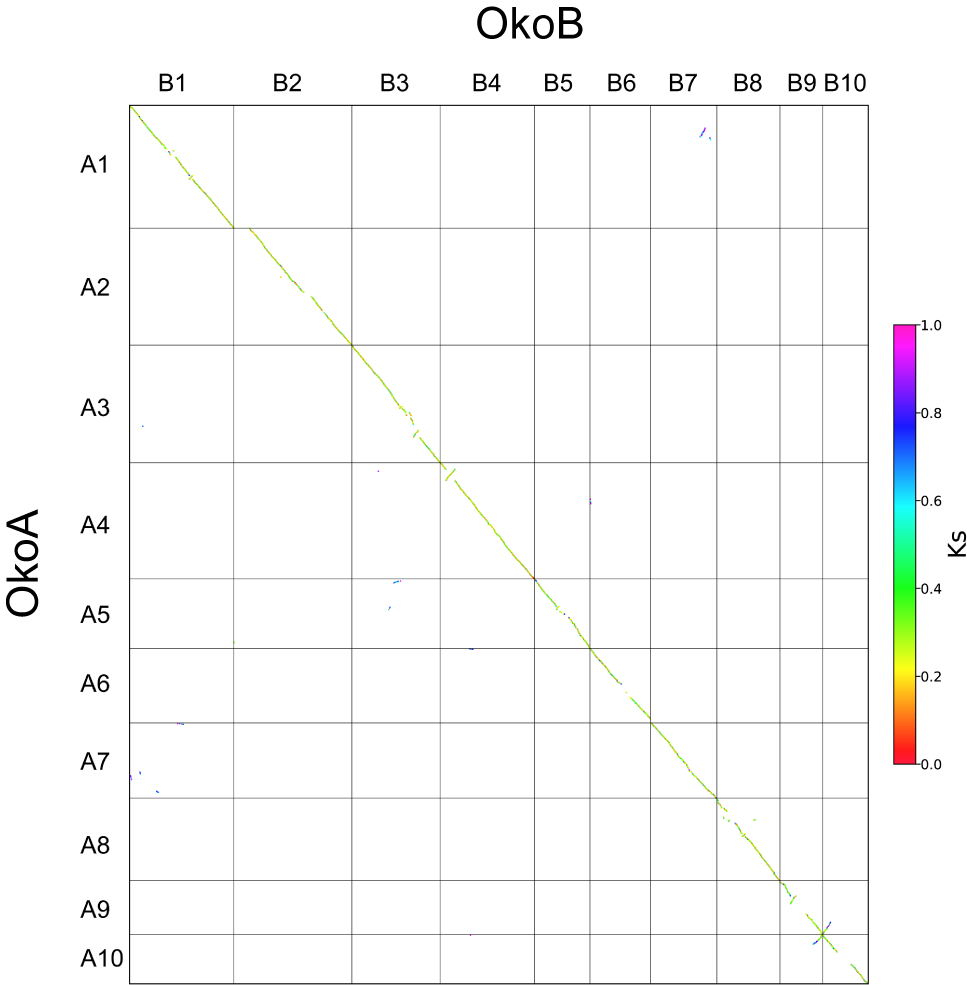
**

Supplementary Figure 5 Overview of *K*s values between the two subgenomes of Oko (*O. kokonorica*).


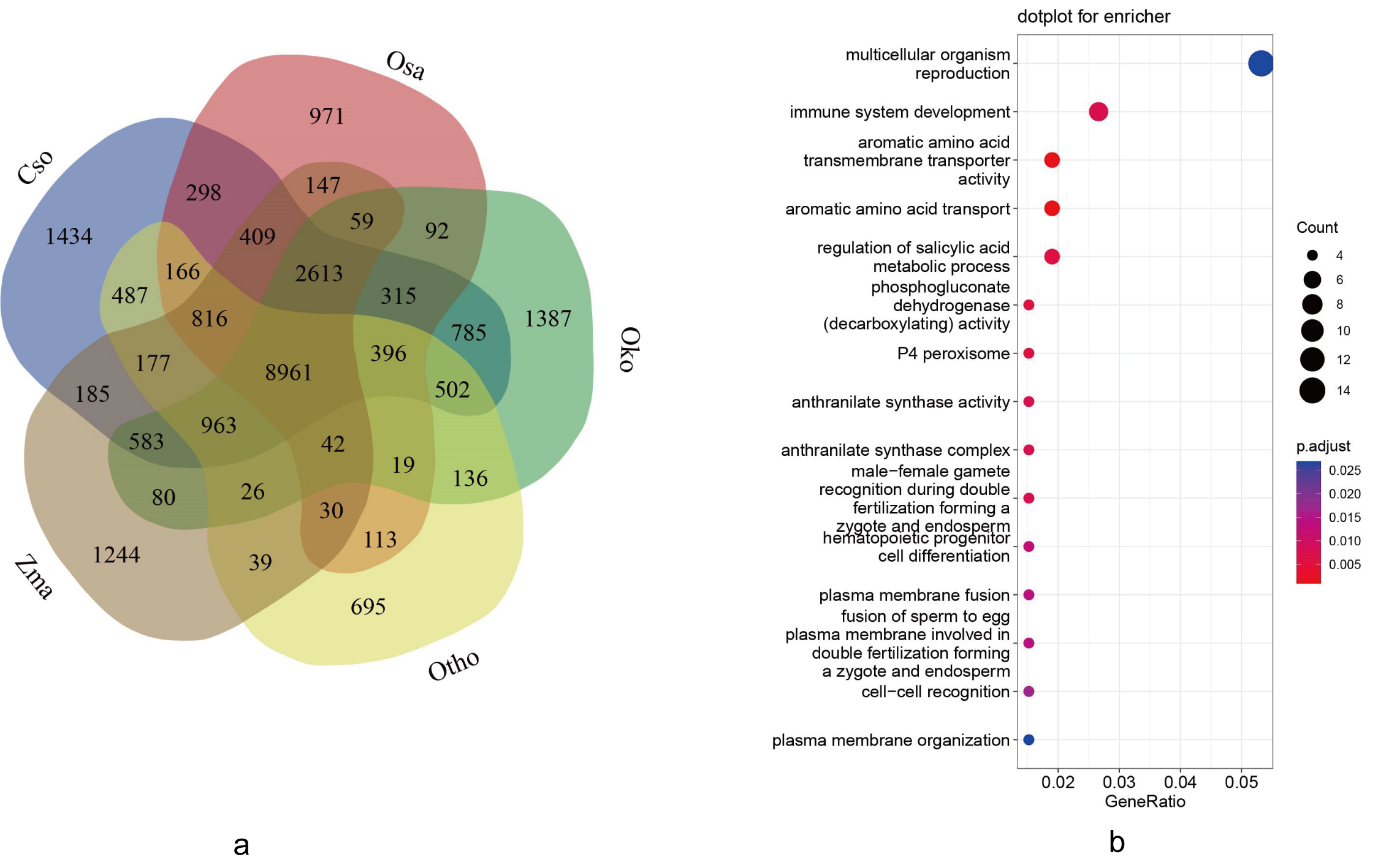


Supplementary Figure 6 a Distribution Venn diagram of orthologous genes shared among five species, Oko (*O. kokonorica*), Cso (*C. songorica*), Osa (*O. sativa*), Otho (*O. thomaeum*), Zma (*Zea mays*). **b** Gene Ontology (GO) enrichment of the unique gene families in *O. kokonorica*. Only the top 15 significant terms are shown. The x-axis (gene ratio) represents the ratio of gene numbers annotated to one GO term and annotated to all GO terms. The color and size of the dots represent the range of the P-value and the number of bract-biased genes mapped to the indicated GO terms, respectively.


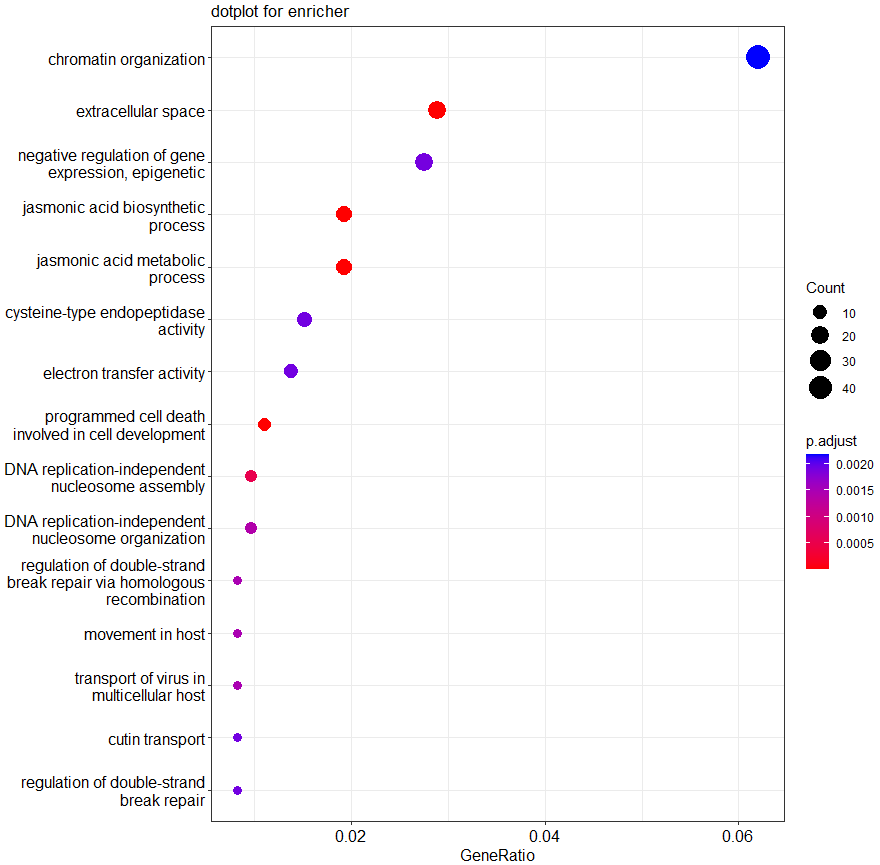


**Supplementary Figure 7** Gene Ontology (GO) enrichment of the expanded gene families in *O. kokonorica*. Only the top 15 significant terms are shown. The x-axis (gene ratio) represents the ratio of gene numbers annotated to one GO term and annotated to all GO terms. The color and size of the dots represent the range of the P-value and the number of bract-biased genes mapped to the indicated GO terms, respectively.


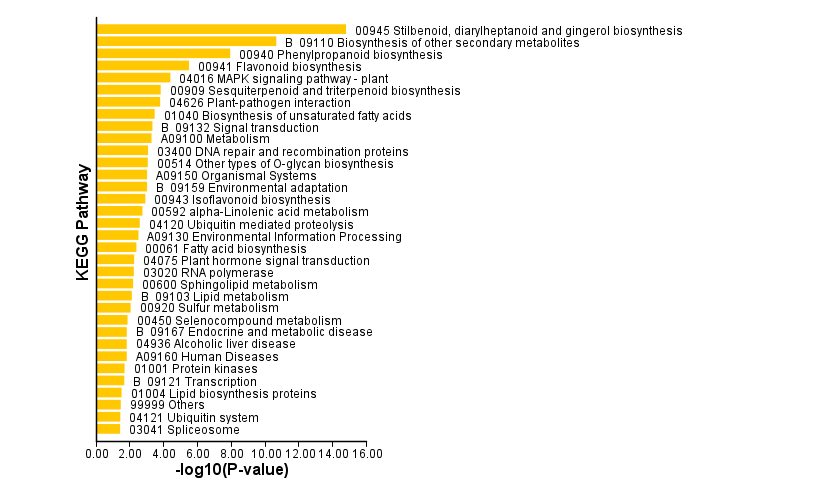


**Supplementary Figure 8** KEGG enrichment of the expanded gene families in *O. kokonorica*.

**
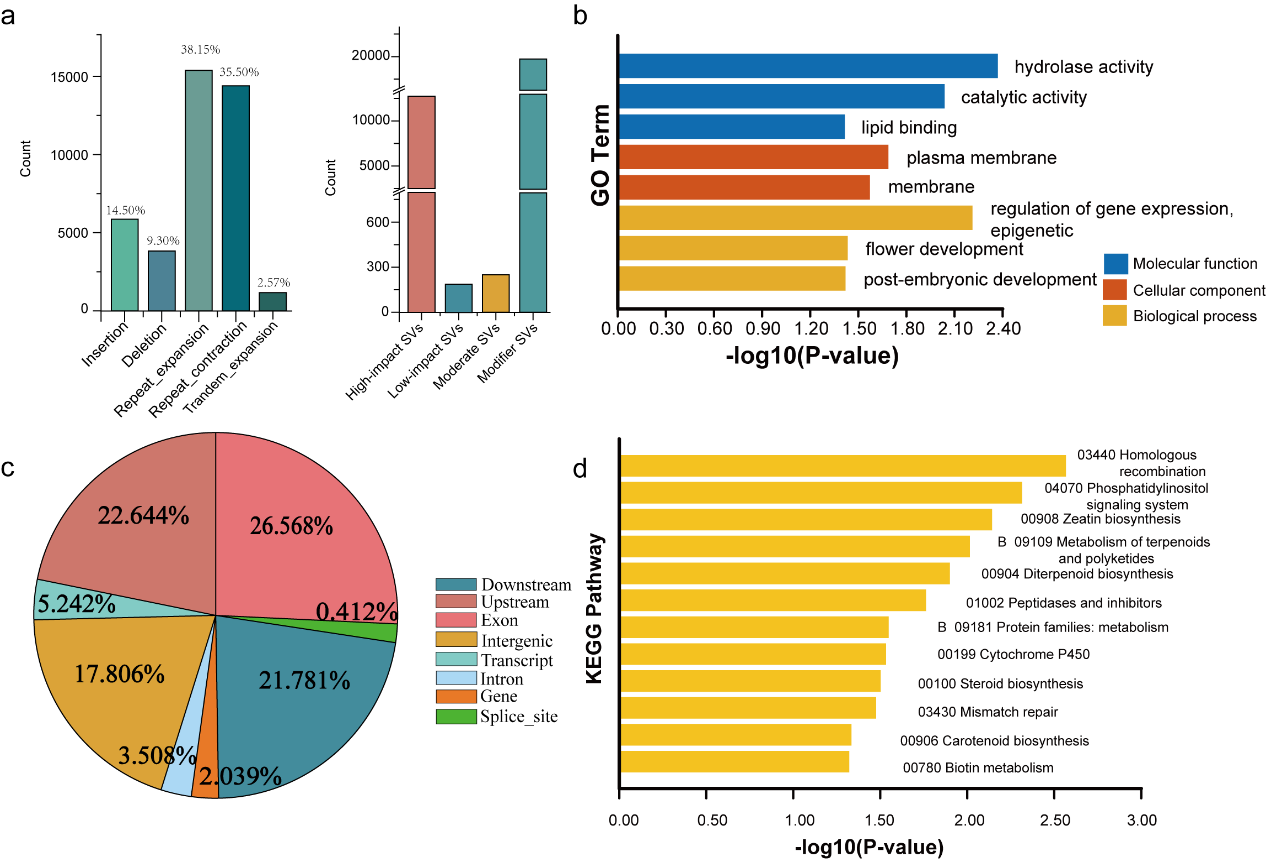
**

**Supplementary Figure 9** SVs identified based on whole-genome alignment in the *O. kokonorica* genome and SV functional enrichment. **a** Number of SVs in *O. kokonoricai*, including deletions, insertions, repeat_expansion, repeat_contraction, and tandem_contraction. Also includes the number of genes in each impacted-by-SV category (High-impact, Medium-impact, Low-impact, and Modifier-impact). **b** Annotation of all SVs categorized using SnpEff based on their positions in the annotated *O. kokonorica* genome. ‘Downstream’ represents SVs located at least 5 kb downstream from a gene; ‘Intergenic’ indicates SVs in the intergenic regions; ‘Splice_site’ indicates a splice variant that changes the 2 bp region at the 3' or 5' end of an intron; ‘Transcipt’ indicates a feature ablation whereby the deleted region includes a transcript feature; ‘Upstream’ indicates SVs located at least 5 kb upstream of a gene. **c** GO enrichment of the highly-impacted-by-SV genes. **d** KEGG enrichment of the highly-impacted-by-SV genes.

**
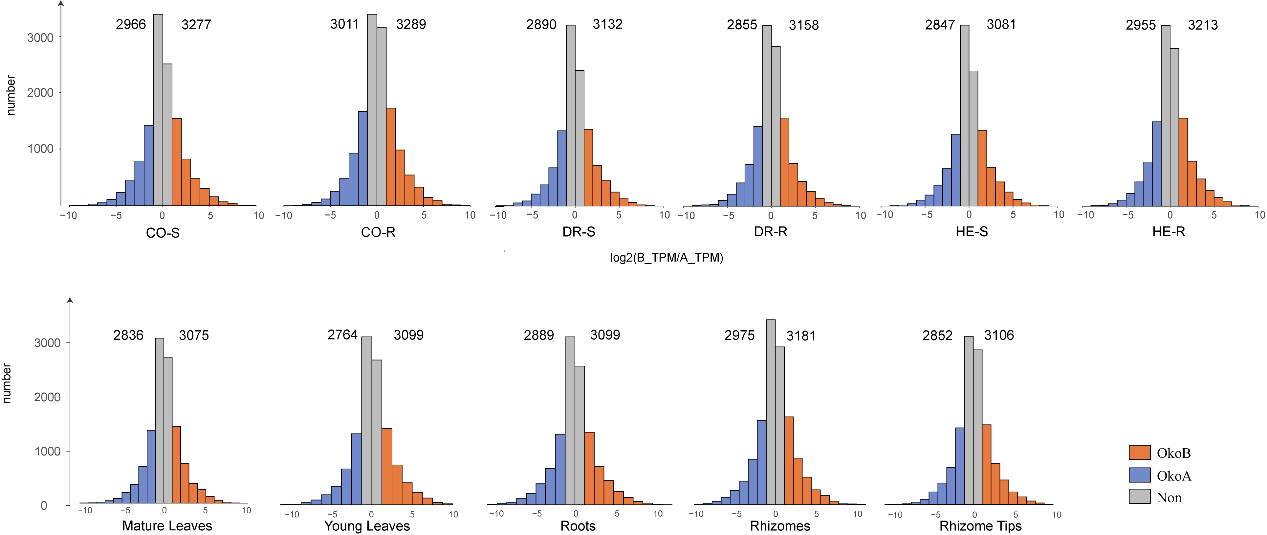
**

**Supplementary Figure 10** Homoeolog expression dominance across three treatments and five tissues in *O. kokonorica*. Histograms of genome-wide expression of syntenic homoeologous genes in various tissues and stress treatments. S, shoot; R, root; CO, cold; DR, drought, HE, heat. N values indicate the number of dominant genes in A subgenome and B subgenome, respectively.


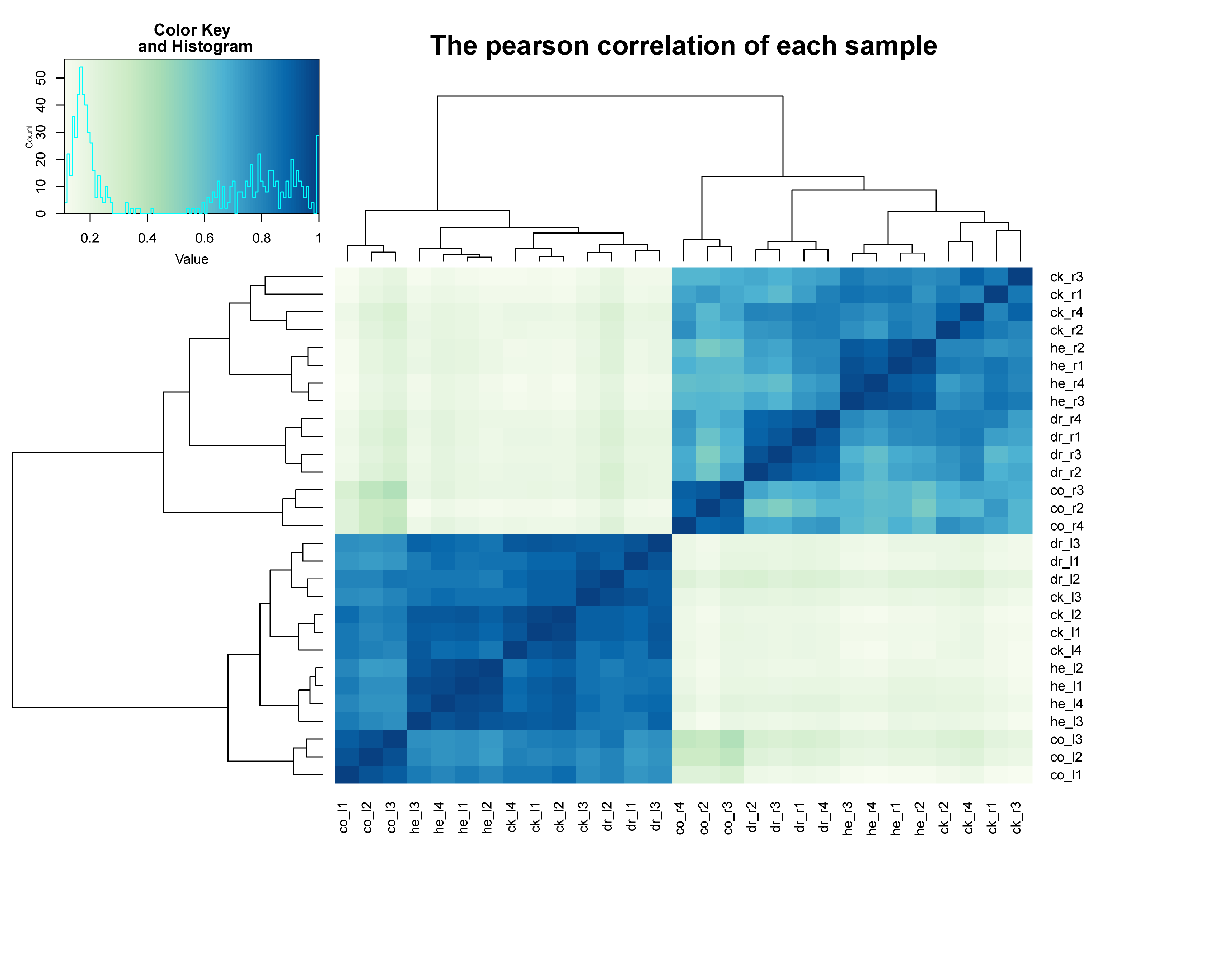


**Supplementary Figure 11** Hierarchical cluster analysis of gene expression under three stress treatments in root and shoot tissues. ck: control check; he: heat treatment; dr: drought treatment; co: cold treatment. r: root; s: shoot. Number means the replicates of treatments.


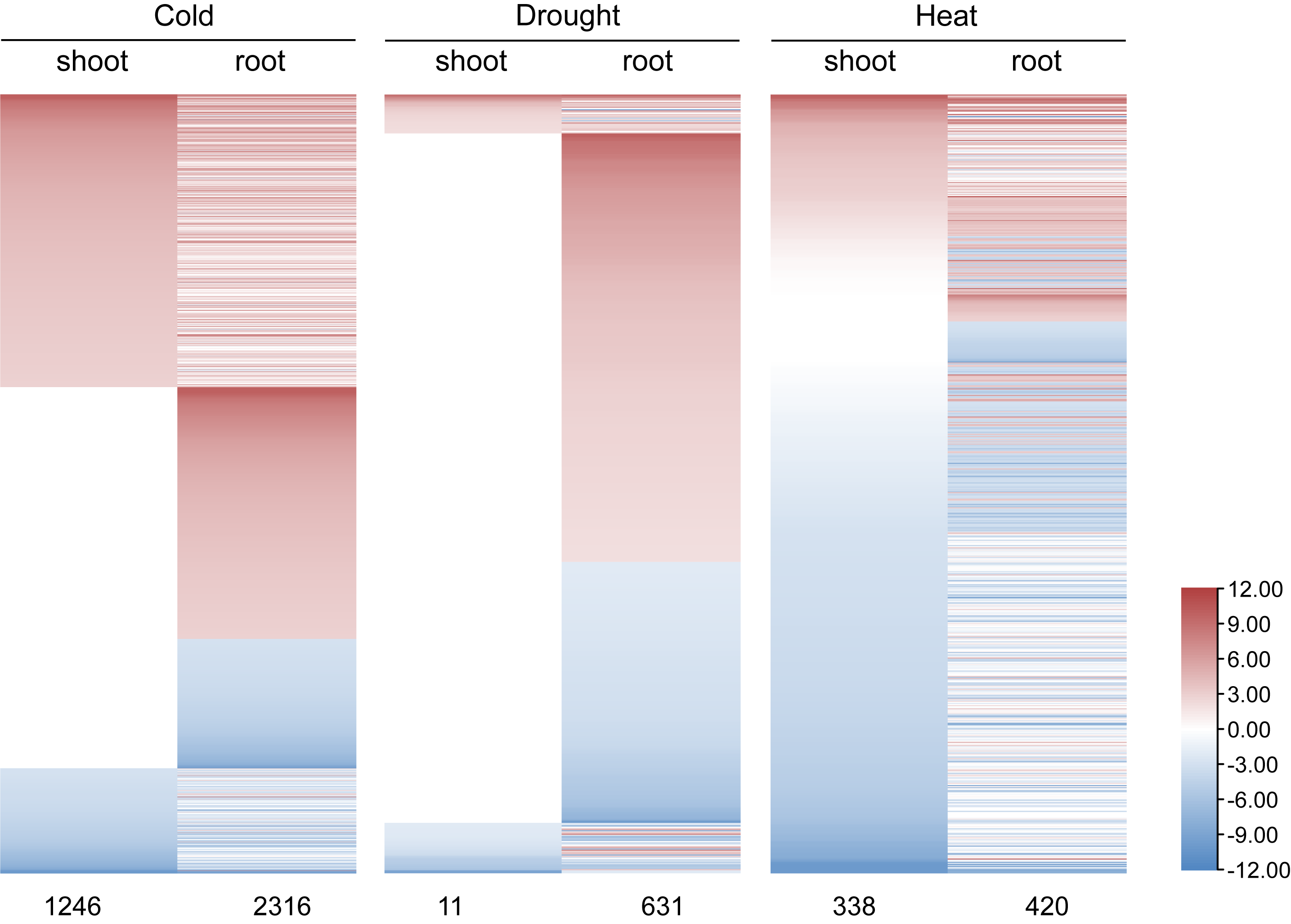


**Supplementary Figure 12** Expression of DEGs identified in roots and shoots under each treatment in *O. kokonorica*. The heatmap was generated from hierarchical cluster analysis of genes. The value under heatmap are the number of DEGs in shoot and root. Each row represents the expression pattern of one gene in the two tissues under each treatment. Blue color indicates downregulation; red color indicates upregulation.


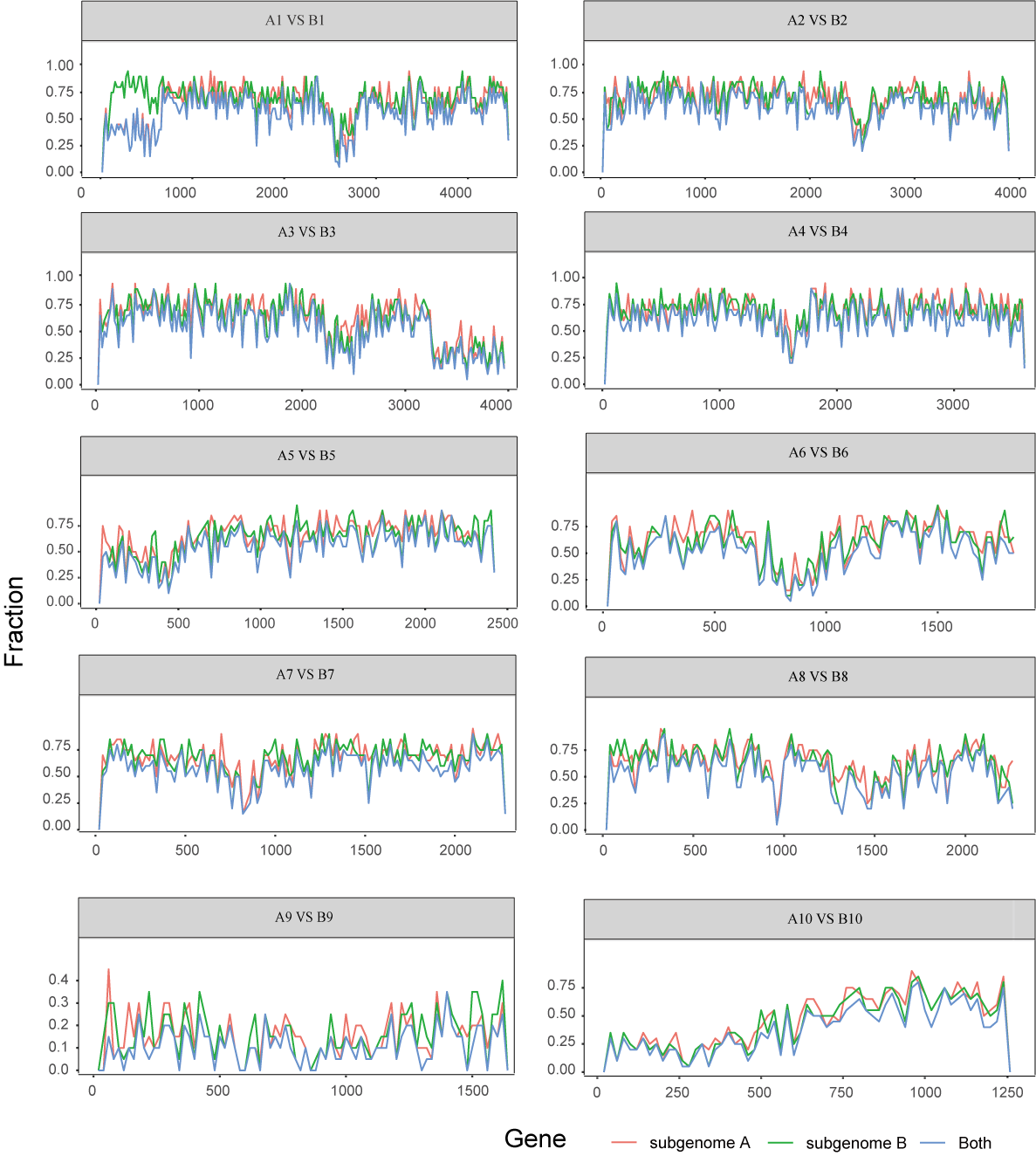


**Supplementary Figure 13** Fractionation in pairs of homoeologous regions in *O. kokonorica*. Average gene retention percentages are shown in a 20-gene window along each subgenome. Retention of genes in subgenomes A, B, and both are shown in red, green, and blue, respectively.

**Supplementary Table 1** Summary of sequence data for *O. kokonorica*.

| Type | Method | Library type | Reads number | Data size (Gb) | Mean read length(bp) | Read N50(bp) |
| --- | --- | --- | --- | --- | --- | --- |
| DNA | Illumina (raw) | Paired | 331851560 | 49.77 | 150 |  |
| DNA | Illumina (clean) | Paired | 236199638 | 35.29 | 149.44 |  |
| DNA | ONT PromethION (raw) | Single | 2793870 | 61.87 | 22146.08 | 23427 |
| DNA | ONT PromethION (consensus) | Single | 253592 | 13.2 | 52044.85 | 53453 |

**Supplementary Table 2** Contig assembly basic statistics of *O. kokonorica*.

| Class | Value |
| --- | --- |
| Contig number | 131 |
| Total length | 555.93 Mb |
| Max contig | 32.15 Mb |
| Min contig | 98.70 Kb |
| N50 | 9.08 Mb |
| L50 | 19 |
| N90 | 1.84 Mb |
| L90 | 70 |
| A | 151356845 (27.25%) |
| T | 151359379 (27.25%) |
| G | 126450565 (22.76%) |
| C | 126324861 (22.74%) |
| CG content | 45.51% |

**Supplementary Table 3** The genome assembly of chromosome-scale based on Hi-C data of *O. kokonorica*.

| **Chromosome** | **Subgenome** | **Chr/Contig Length (bp)** | **Contig Number** | **GC ratio** | **Gene Number** |
| --- | --- | --- | --- | --- | --- |
| LG01 | B1 | 39'176'818 | 14 | 0.4554 | 3894 |
| LG02 | A2 | 32'623'288 | 5 | 0.4565 | 3376 |
| LG03 | B2 | 32'384'499 | 6 | 0.4571 | 3439 |
| LG04 | A1 | 32'154'167 | 1 | 0.4561 | 3212 |
| LG05 | A3 | 32'008'803 | 4 | 0.4564 | 3232 |
| LG06 | A4 | 31'203'026 | 2 | 0.456 | 3192 |
| LG07 | B3 | 29'349'415 | 7 | 0.4551 | 2917 |
| LG08 | B8 | 29'267'593 | 13 | 0.4514 | 1998 |
| LG09 | B4 | 28'443'040 | 5 | 0.4548 | 3110 |
| LG10 | A6 | 26'689'284 | 5 | 0.4553 | 1914 |
| LG11 | A8 | 26'479'511 | 4 | 0.4559 | 2045 |
| LG12 | B5 | 25'272'558 | 5 | 0.457 | 2085 |
| LG13 | B7 | 24'833'469 | 4 | 0.4564 | 2187 |
| LG14 | B6 | 24'762'606 | 17 | 0.454 | 1837 |
| LG15 | A7 | 24'417'713 | 6 | 0.4557 | 2065 |
| LG16 | A5 | 24'078'586 | 10 | 0.459 | 2266 |
| LG17 | B10 | 23'863'308 | 4 | 0.4529 | 1507 |
| LG18 | A9 | 23'247'314 | 5 | 0.451 | 1483 |
| LG19 | B9 | 23'246'382 | 6 | 0.4518 | 1408 |
| LG20 | A10 | 21'353'989 | 4 | 0.4515 | 1354 |
| Contig1 | - | 389'191 | 1 | 0.4553 | 26 |
| Contig2 | - | 249'346 | 1 | 0.3969 | 0 |
| Contig3 | - | 184'884 | 1 | 0.437 | 29 |
| Contig4 | - | 167'421 | 1 | 0.438 | 22 |
| Contig5 | - | 100'000 | 1 | 0.438 | 0 |
| Total | - | 555'946'211 | 132 | 0.4551 | 48598 |

**Supplementary Table 4** Statistics of short read alignment.

| Mapped Data (Mb) | 34918.34 |
| --- | --- |
| Fraction of Mapped Data (Mb) | 98.93% |
| Fraction of Properly paired Mapped | 91.37% |
| Fraction of MapQ (>20) reads in mapped reads | 86.93% |
| Average depth | 61.89 |
| Average depth (delete dup) | 53.94 |
| Coverage (>0x) | 95.19% |
| Coverage (>=4x) | 94.25% |
| Coverage (>=10x) | 93.06% |
| Genomic region covered (>0x) | 100% |

“Average depth (delete dup)” means the average depth after deleting haplotigs and overlaps in an assembly based on read depth.

**Supplementary Table 5** Statistics of repetitive elements.

| Repetitive element type | Length(bp) of genome | Percentage (%) of genome |
| --- | --- | --- |
| **DNA** | 49570403 | 8.916 |
| DNA_CMC-EnSpm | 18312349 | 3.294 |
| DNA_other | 31420524 | 5.652 |
| **LINE** | 19763955 | 3.555 |
| LINE_L1 | 18102005 | 3.256 |
| LINE_L2 | 105492 | 0.019 |
| LINE_other | 1939976 | 0.349 |
| **LTR** | 200240290 | 36.018 |
| LTR_Copia | 40564538 | 7.296 |
| LTR_Gypsy | 159627056 | 28.713 |
| LTR_other | 2106964 | 0.379 |
| Low_complexity | 340402 | 0.061 |
| **SINE** | 1204928 | 0.217 |
| Satellite | 3341573 | 0.601 |
| Simple_repeat | 34083221 | 6.131 |
| Small_RNA | 229278 | 0.041 |
| Unclassified_RC/Helitron | 4188305 | 0.753 |
| Unclassified_Retroposon/L1 | 47897 | 0.009 |
| Unclassified_Unknown | 51043787 | 9.181 |
| **Total** | 326963690 | 58.81% |

**Supplementary Table 6** Summary of predicted protein-coding gene annotations and their supporting evidence types.

| Gene set | | | Total Genes Predicted | | Average Gene Length (bp) | | Average CDS Length (bp) | | Average Exons per Gene | | Average Exon Length (bp) | | Average Intron Length (bp) | |
| --- | --- | --- | --- | --- | --- | --- | --- | --- | --- | --- | --- | --- | --- | --- |
| *De novo* | AUGUSTUS | 56'236 | | 3'844.62 | | 1'466.93 | | 6.65 | | 220.53 | | 319.24 | |  |
|  | GENSCAN | 46'297 | | 5'066.09 | | 672.33 | | 3.24 | | 207.07 | | 1955.48 | |  |
| Homolog | *A. thaliana* | 30'098 | | 3'318.29 | | 1276.22 | | 5.87 | | 217.38 | | 419.24 | |  |
|  | *O. sativa* | 38'621 | | 3'165.48 | | 1305.03 | | 5.43 | | 240.14 | | 419.55 | |  |
|  | *E. tef* | 26'138 | | 3'655.92 | | 1195.63 | | 6.02 | | 198.55 | | 489.94 | |  |
|  | *P. persica* | 31'516 | | 3'310.96 | | 1313.16 | | 5.72 | | 229.49 | | 423.08 | |  |
|  | *S. bicolor* | 39'205 | | 3'068.44 | | 1237.28 | | 5.34 | | 231.46 | | 421.38 | |  |
|  | *Z. mays* | 40'765 | | 3'004.40 | | 1210.15 | | 5.22 | | 231.57 | | 424.6 | |  |
|  | *E. curvula* | 38'054 | | 3'336.07 | | 1248.01 | | 5.56 | | 224.1 | | 457.01 | |  |
| RNA_seq |  | 63'582 | | 4'212.97 | | 953.31 | | 4.27 | | 223.02 | | 541.07 | |  |
| EVM |  | 48'598 | | 4'327.40 | | 1'346.61 | | 6.17 | | 217.92 | | 575.53 | |  |

**Supplementary Table 7** Comparison of gene annotation.

| Species | Total_Genes | Average Gene Length (bp) | Average CDS Length (bp) | Average Exons per Gene | Average Exon Length (bp) | Average Intron Length (bp) |
| --- | --- | --- | --- | --- | --- | --- |
| OkoA | 24139 | 4272.47 | 1340.47 | 6.16 | 217.73 | 568.59 |
| OkoB | 24459 | 4381.62 | 1352.69 | 6.2 | 218.13 | 582.34 |
| CsoA | 27382 | 2700.53 | 1141.22 | 4.81 | 237.02 | 408.74 |
| CsoB | 27936 | 2692.79 | 1143.01 | 4.8 | 238.44 | 408.51 |
| *O.sativa* | 42579 | 3394.78 | 1421.613 | 5.61 | 253.36 | 428.8 |
| *Z. mays* | 39591 | 4155.38 | 1446.86 | 7.03 | 205.85 | 581.85 |
| *S. bicolor* | 34118 | 3711.97 | 1228.18 | 5.09 | 241.13 | 468.87 |

**Supplementary Table 8** BUSCO statistics of genome assembly and annotation of *O. kokonorica*.

| Class | Genome | | | Gene model | |
| --- | --- | --- | --- | --- | --- |
|  | Count | Rate | Count | | Rate |
| Complete BUSCOs(C) | 1576 | 97.6 | 1463 | | 90.6 |
| Complete and single-copy BUSCOs(S) | 912 | 56.5 | 924 | | 57.2 |
| Complete and duplicated BUSCOs(D) | 664 | 41.1 | 539 | | 33.4 |
| Fragmented BUSCOs(F) | 14 | 0.9 | 55 | | 3.4 |
| Missing BUSCOs(M) | 24 | 1.5 | 96 | | 6.0 |
| Total BUSCO groups searched | 1614 | 100 | 1614 | | 100 |

**Supplementary Table 9** Functional annotation of the predicted genes of *O. kokonorica*.

|  | Database | Gene number | Percent |
| --- | --- | --- | --- |
| Total |  | 48598 |  |
|  | InterPro | 43243 | 88.98% |
|  | GO | 23156 | 47.65% |
|  | KEGG | 16159 | 33.25% |
|  | SwissProt | 28668 | 58.99% |
|  | KOG | 31321 | 64.45% |
| Annotated |  | 44024 | 90.59% |
| Unannotated |  | 4574 | 9.41% |

**Supplementary Table 10** The ncRNA annotation of *O. kokonorica*.

| Software | Type | Number | Average length |
| --- | --- | --- | --- |
| INFERNAL | miRNA | 231 | 130.61 |
|  | snRNA | 1012 | 123.29 |
| tRNAscan-SE | tRNA | 903 | 73.88 |
| BLAST | rRNA | 183 | 304.34 |

**Supplementary Table 11** Summary of gene family clustering.

| Species | | Total_genes | | Genes_in_families | | Unclustered_genes | | Families | | Unique_families | | Genes_per_family | Maximum_gene_family_size |
| --- | --- | --- | --- | --- | --- | --- | --- | --- | --- | --- | --- | --- | --- |
| Aco | 21538 | | 19306 | | 2232 | | 12539 | | 276 | | 1.53967621 | | 138 |
| Ath | 27525 | | 23277 | | 4248 | | 12075 | | 1117 | | 1.927701863 | | 146 |
| Bdi | 25374 | | 23314 | | 2060 | | 16038 | | 193 | | 1.453672528 | | 64 |
| CsoA | 27382 | | 22611 | | 4771 | | 16792 | | 92 | | 1.346534064 | | 40 |
| CsoB | 27936 | | 23067 | | 4869 | | 16921 | | 88 | | 1.363217304 | | 40 |
| DexA | 28576 | | 21817 | | 6759 | | 15460 | | 236 | | 1.411190168 | | 41 |
| EteA | 34032 | | 27487 | | 6545 | | 19415 | | 216 | | 1.41576101 | | 88 |
| EteB | 32255 | | 26651 | | 5604 | | 19237 | | 160 | | 1.385403129 | | 55 |
| Hvu | 39734 | | 31729 | | 8005 | | 16571 | | 1490 | | 1.914730553 | | 218 |
| Mac | 30495 | | 27804 | | 2691 | | 12710 | | 492 | | 2.187568843 | | 123 |
| Osa | 28271 | | 21943 | | 6328 | | 15447 | | 302 | | 1.420534732 | | 67 |
| OkoA | 24139 | | 16922 | | 7217 | | 13014 | | 253 | | 1.300291993 | | 32 |
| OkoB | 24459 | | 17097 | | 7362 | | 12889 | | 294 | | 1.326479944 | | 36 |
| Otho | 24459 | | 17253 | | 7206 | | 13568 | | 236 | | 1.271594929 | | 18 |
| Peq | 18749 | | 16215 | | 2534 | | 10992 | | 316 | | 1.475163755 | | 102 |
| Pha | 26323 | | 24830 | | 1493 | | 16914 | | 115 | | 1.468014662 | | 85 |
| Sbi | 28062 | | 26014 | | 2048 | | 17082 | | 223 | | 1.522889591 | | 77 |
| Sit | 27222 | | 25510 | | 1712 | | 16892 | | 111 | | 1.510182335 | | 64 |
| Zma | 34081 | | 30334 | | 3747 | | 16374 | | 636 | | 1.852571149 | | 106 |

**Supplementary Table 12** Statistics of the expanded and contracted gene families.

| Species | Expanded | Significantly Expanded | Genes gained | genes/expansion | Contracted | Significantly Contracted | Genes lost | genes/contraction | No.change | Avg.Expansion |
| --- | --- | --- | --- | --- | --- | --- | --- | --- | --- | --- |
| Aco | 1360 | 241 | 2407 | 1.77 | 14851 | 152 | 15127 | 1.02 | 10360 | -0.478717 |
| Ath | 2091 | 431 | 3733 | 1.79 | 16156 | 145 | 16412 | 1.02 | 8324 | -0.477174 |
| Bdi | 983 | 844 | 1539 | 1.57 | 1906 | 64 | 2033 | 1.07 | 23682 | -0.018592 |
| Csoa | 1234 | 1148 | 1563 | 1.27 | 1645 | 1575 | 1658 | 1.01 | 23692 | -0.003575 |
| Csob | 1350 | 1285 | 1756 | 1.3 | 1491 | 1430 | 1500 | 1.01 | 23730 | 0.009635 |
| Dexa | 1065 | 973 | 1442 | 1.35 | 5123 | 4188 | 5719 | 1.12 | 20383 | -0.160965 |
| Etea | 1185 | 1141 | 1857 | 1.57 | 1295 | 1228 | 1306 | 1.01 | 24091 | 0.020737 |
| Eteb | 969 | 941 | 1308 | 1.35 | 1435 | 1368 | 1448 | 1.01 | 24167 | -0.005269 |
| Hvu | 2372 | 2109 | 6040 | 2.55 | 2880 | 89 | 2990 | 1.04 | 21319 | 0.114787 |
| Mac | 4872 | 1906 | 9102 | 1.87 | 14460 | 46 | 14545 | 1.01 | 7239 | -0.204847 |
| Okoa | 495 | 482 | 653 | 1.32 | 6400 | 5618 | 6848 | 1.07 | 19676 | -0.233149 |
| Okob | 549 | 526 | 723 | 1.32 | 6556 | 5845 | 6949 | 1.06 | 19466 | -0.234316 |
| Osa | 947 | 729 | 1479 | 1.56 | 4165 | 136 | 4368 | 1.05 | 21459 | -0.108728 |
| Otho | 764 | 680 | 896 | 1.17 | 8577 | 7469 | 9498 | 1.11 | 17230 | -0.323736 |
| Peq | 849 | 127 | 1442 | 1.7 | 17149 | 296 | 17698 | 1.03 | 8573 | -0.611795 |
| Pha | 746 | 681 | 1316 | 1.76 | 1474 | 1187 | 1558 | 1.06 | 24351 | -0.009108 |
| Sbi | 1054 | 998 | 2102 | 1.99 | 1141 | 1014 | 1161 | 1.02 | 24376 | 0.035415 |
| Sit | 1142 | 1062 | 2008 | 1.76 | 1430 | 1183 | 1478 | 1.03 | 23999 | 0.019947 |
| Zma | 3962 | 3560 | 6543 | 1.65 | 2329 | 2220 | 2518 | 1.08 | 20280 | 0.151481 |

**Supplementary Table 13** KEGG enrichment analysis of the expanded gene family in *O. kokonorica*.

| **Term Name** | **MainClass** | **Gene Hits In Selected Set** | **Gene Hits In Background** | **p-value** | **enrichFactor** | **corrected p-value (BH method)** |
| --- | --- | --- | --- | --- | --- | --- |
| 00945 Stilbenoid, diarylheptanoid and gingerol biosynthesis | A09100 Metabolism | 28 | 84 | 1.67E-15 | 6.16604862 | 2.50E-13 |
| B09110 Biosynthesis of other secondary metabolites | A09100 Metabolism | 69 | 542 | 2.20E-11 | 2.354930008 | 1.65E-09 |
| 00940 Phenylpropanoid biosynthesis | A09100 Metabolism | 46 | 344 | 1.17E-08 | 2.473589272 | 5.84E-07 |
| 00941 Flavonoid biosynthesis | A09100 Metabolism | 15 | 69 | 3.17E-06 | 4.021336056 | 1.19E-04 |
| 04016 MAPK signaling pathway - plant | A09130 Environmental Information Processing | 38 | 353 | 4.01E-05 | 1.991301821 | 0.001002623 |
| 00909 Sesquiterpenoid and triterpenoid biosynthesis | A09100 Metabolism | 7 | 23 | 1.50E-04 | 5.629870479 | 0.002817725 |
| 04626 Plant-pathogen interaction | A09150 Organismal Systems | 40 | 404 | 1.61E-04 | 1.83149959 | 0.00267892 |
| 01040 Biosynthesis of unsaturated fatty acids | A09100 Metabolism | 10 | 51 | 3.41E-04 | 3.627087423 | 0.005119113 |
| B09132 Signal transduction | A09130 Environmental Information Processing | 66 | 806 | 4.61E-04 | 1.51473651 | 0.00628071 |
| A09100 Metabolism | A09100 Metabolism | 264 | 4114 | 5.22E-04 | 1.187046793 | 0.006523397 |
| 03400 DNA repair and recombination proteins | A09180 Brite Hierarchies | 42 | 467 | 8.39E-04 | 1.66364481 | 0.00967616 |
| 00514 Other types of O-glycan biosynthesis | A09100 Metabolism | 6 | 22 | 8.66E-04 | 5.044948871 | 0.00928012 |
| A09150 Organismal Systems | A09150 Organismal Systems | 44 | 499 | 9.54E-04 | 1.631099034 | 0.009543724 |
| B09159 Environmental adaptation | A09150 Organismal Systems | 44 | 499 | 9.54E-04 | 1.631099034 | 0.009543724 |
| 00943 Isoflavonoid biosynthesis | A09100 Metabolism | 5 | 16 | 0.001208205 | 5.780670581 | 0.010660632 |
| 00592 alpha-Linolenic acid metabolism | A09100 Metabolism | 12 | 84 | 0.001792823 | 2.642592266 | 0.014940193 |
| 04120 Ubiquitin mediated proteolysis | A09120 Genetic Information Processing | 24 | 239 | 0.002594615 | 1.857554396 | 0.020483803 |
| A09130 Environmental Information Processing | A09130 Environmental Information Processing | 66 | 869 | 0.003084725 | 1.40492247 | 0.023135434 |
| 00061 Fatty acid biosynthesis | A09100 Metabolism | 11 | 81 | 0.004093644 | 2.512093882 | 0.029240317 |
| 04075 Plant hormone signal transduction | A09130 Environmental Information Processing | 39 | 472 | 0.005548278 | 1.528448493 | 0.037829166 |
| 03020 RNA polymerase | A09120 Genetic Information Processing | 9 | 62 | 0.005820513 | 2.685214721 | 0.037959868 |
| 00600 Sphingolipid metabolism | A09100 Metabolism | 8 | 52 | 0.006403867 | 2.845868594 | 0.040024169 |
| B09103 Lipid metabolism | A09100 Metabolism | 53 | 698 | 0.00764671 | 1.404587006 | 0.045880263 |

**Supplementary Table 14** The 20 AMGs with differential expression (two-fold change) in CLs compared with that in CHs in *C. songorica*, and their orthologs in *O. kokonorica*.

| Cluster | Cso | CH | CL | Ortholog in Oko |
| --- | --- | --- | --- | --- |
| A | CsB800132 | 91.281166 | 205.291458 | Oko025226 |
|  | CsB800713 | 0.888216 | 4.202277 | Oko024740 |
|  | CsB400262 | 3.12187 | 14.931536 | Oko028162 |
| B | CsB701351 | 51.930883 | 16.791985 | Oko035214 |
|  | CsA303529 | 122.30129 | 34.328442 | Oko017066 |
|  | CsB303586 | 56.647821 | 23.965889 | Lost |
| C | CsB700612 | 98.847771 | 274.858643 | Oko036040 |
|  | CsA701717 | 190.98615 | 387.922729 | Oko038894 |
|  | CsB501322 | 7.866383 | 39.071678 | Oko009572 |
|  | CsA201468 | 7.687432 | 18.309391 | Oko006142 |
|  | CsA701471 | 6.287592 | 16.746031 | Oko025829 |
|  | CsA402979 | 93.289696 | 250.786942 | Oko019761 |
| D | CsB802246 | 9.095182 | 20.715839 | Oko003621 |
|  | CsA100369 | 4.241242 | 15.576239 | Oko010971 |
|  | CsA1000989 | 66.82682 | 242.153793 | Oko048186 |
|  | CsB1000971 | 59.519455 | 170.403992 | Lost |
|  | CsB502247 | 35.412518 | 143.113159 | Oko010346 |
|  | CsA200526 | 37.867458 | 182.161728 | Oko006900 |
| E | CsB600279 | 34.255989 | 102.434944 | Lost |
|  | CsA400788 | 77.538872 | 235.813538 | Oko017668 |

**Supplementary Table 15** The detailed SVs in the *BOP* orthologous pairs between the two species.

| *BOP* gene in *O. kokonorica* | *BOP* gene in *C. songorica* | Type | Position | Length | Distance |
| --- | --- | --- | --- | --- | --- |
| Oko007168 | CsA200175 | INS | upstream | 230 | 1674 |
|  |  | DEL | downstream | 86 | 1711 |
| Oko010623 | CsB502591 | INS | upstream | 106 | 669 |
|  |  | INS | downstream | 226 | 223 |
| Oko048400 | CsA1001253 | INS | upstream | 82 | 635 |
|  |  | DEL | upstream | 231 | 1249 |
|  |  | INS | downstream | 69 | 1889 |
|  |  | INS | downstream | 916 | 2105 |
| Oko042875 | CsB1001283 | INS | intron | 363 | - |
|  |  | DEL | exon | 73 | - |
|  |  | DEL | upstream | 405 | 476 |
|  |  | INS | downstream | 62 | 1238 |
|  |  | DEL | downstream | 332 | 1741 |
| Oko047059 | CsB900242 | DEL | intron | 120 | - |
|  |  | DEL | upstream | 162 | 1766 |
| Oko044410/Oko044411 | CsA901337 | DEL | downstream | 479 | 12 |
|  |  | DEL | downstream | 795 | 694 |

**Supplementary Table 16** Comparison of genome assembly among four species.

| Species | Genome size (Mb) | Gene number | Repetitive content (%) |
| --- | --- | --- | --- |
| *O. kokonorica* | 556 | 48,598 | 58.8 |
| *C. songorica* | 540 | 54,383 | 42.0 |
| *E. tef* | 578 | 50,459 | 24.6 |
| *L. chinensis* | 416 | 68,255 | 26.5 |
