# Supplementary material for: A genome assembly for Orinus kokonorica provides insights into the origin, adaptive evolution and further diversification of two closely related grass genera
Source: Commun Biol. 2023 Dec 2;6:1223. doi: 10.1038/s42003-023-05620-5 (PMC10693610; doi:10.1038/s42003-023-05620-5)
Supplement: Supplementary file 3 — Description of Additional Supplementary Files [file 42003_2023_5620_MOESM3_ESM.docx]

Description of Additional Supplementary Files

**File name:** Supplementary Data 1

**Description:** The source data behind the Fig. 2b.

**File name:** Supplementary Data 2

**Description:** Gene ontology (GO) enrichment analysis of the expanded gene families in O. kokonorica.

**File name:** Supplementary Data 3

**Description:** The expression of orthologous DEG pairs in O. kokonorica and C. songorica under cold stress and the source data behind the Fig. 3b.

**File name:** Supplementary Data 4

**Description:** The expression of orthologous DEG pairs in O. kokonorica and C. songorica under drought stress and the source data behind the Fig. 3b.

**File name:** Supplementary Data 5

**Description:** The expression of orthologous DEG pairs in O. kokonorica and C. songorica under heat stress and the source data behind the Fig. 3b.
